# Mining precise cause and effect rules in large time series data of socio-economic indicators

**DOI:** 10.1186/s40064-016-3292-0

**Published:** 2016-09-21

**Authors:** Swati Hira, P. S. Deshpande

**Affiliations:** Department of Computer Science and Engineering, Visvesvaraya National Institute of Technology, 440010 Nagpur, India

**Keywords:** Data mining, Cause–effect relationships, Causality, Temporal association, Temporal odds ratio

## Abstract

Discovery of cause–effect relationships, particularly in large databases of time-series is challenging because of continuous data of different characteristics and complex lagged relationships. In this paper, we have proposed a novel approach, to extract cause–effect relationships in large time series data set of socioeconomic indicators. The method enhances the scope of relationship discovery to cause–effect relationships by identifying multiple causal structures such as binary, transitive, many to one and cyclic. We use temporal association and temporal odds ratio to exclude noncausal association and to ensure the high reliability of discovered causal rules. We assess the method with both synthetic and real-world datasets. Our proposed method will help to build quantitative models to analyze socioeconomic processes by generating a precise cause–effect relationship between different economic indicators. The outcome shows that the proposed method can effectively discover existing causality structure in large time series databases.

## Background

**Fig. 1:**
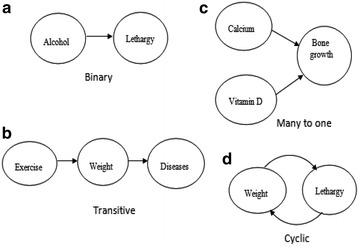
Causal relationships

A system such as mechanical, biological or social-economic system consists of independent components. These components influence one another to maintain their activity for the existence of a system in order to achieve the goal of the system. The system changes behavior when a component is changed or removed significantly. This motivates us to find the reason or cause behind fault and discover the cause parameters in explaining the interactions among the components of a system or process. The causal discovery indicates not only that the indicators are correlated, but also how changing a cause variable is expected to induce a change in an effect variable. For example, with analyzed cause–effect relationships, we can predict potential effects before taking any actions (causes), which is useful in preventing inaccurate decision or policy making in the social-economical system. Time series data can be used to extract delayed relationship between two variables, for example, “CO2 emission occurring at a place might cause air pollution at another place after some delay”. These lagged relationships signify the time lag between the cause–effect parameters. Identifying lagged relationships between socioeconomic processes is challenging due to the presence of various complex dependencies in the data. This dependency among the various parameters has enabled us to identify relationships among different domain parameters in time series data (Madsen 2007; Geweke 1984). The cause–effect relationship for time series prediction is a step towards extracting the various existing causal relations between different domain, such as employment, education, agriculture and rural development etc. Causal discovery has been used in various fields with great success as bioinformatics (Needham et al. 2007), biology (Shipley 2002), earth sciences, etc. to identify protein interactions (Sachs et al. 2005; Chen et al. 2010), gene regulatory networks (Pinna et al. 2010; Friedman et al. 2007) and to study atmospheric teleconnections (Chu et al. 2005). It has also emerged in economics and social sciences (Spirtes et al. 2000; Neapolitan 2004) such as to improve the economic development (Easterly and Levine 2003) and growth (Asafu-Adjaye 2000) of a country and to study the impact of climate change (Ebert-Uphoff and Deng 2014; Deng and Ebert-Uphoff 2014). Before describing the proposed method to extract various causal rules, we explain the following example (Fig. 1) to show the motivation of our research.

Suppose we have set of indicators such as exercise, weight, diseases, calcium, alcohol, and bone growth etc. Various causal relationships can exists among them. An indicator may affect other instantly or after some time. For example, if a person takes alcohol he may feel a lack of energy (lethargy) instantly or after some time (Fig. 1a). If he takes alcohol frequently, the changes can be observed and it can be concluded that alcohol is one of the causes behind tiredness. We could identify the time between alcohol was taken and occurrence of lethargy and can also identify the amount of alcohol dose tends to cause the lethargy. More relationship like transitive can be analyzed between set of indicators (shown in Fig. 1), such as lack of exercise increases weight, which increases the chance of diseases (Fig. 1b, c). Many to one, shows the relationship such as if a person is taking the proper dose of calcium and vitamin D, it will help in bone growth i.e. bone growth requires both calcium and vitamin D. Figure 1d describes the cyclic relationship mean properties affecting each other in a cyclic manner, for example, lethargy increases weight which in turn also increases lethargy. These extracted relationships are referred as binary, transitive, many to one and cyclic respectively.

In this paper, we have proposed a method to extract various causal relationships as binary, transitive, many to one and cyclic with properties such as time required to occur an effect (as lag value), rate of change (of both cause and effect parameter) and strength of a relationship without using statistical information.

## Related work and contributions

The common way to identify cause–effect relationships is to plan randomized controlled experiments, which is generally expensive and unattainable with a huge number of parameters. Therefore, much concentration is needed to discover cause–effect relationships from increased growth of the huge amount of observational data. Discovery of cause–effect relationships in large observational data is a demandable task. Pearl and Verma (1991) suggested a framework that discovered causal structures from connected conditional independence, based on that some techniques have been developed to identify the causal relationships. However, still it cannot discover causal structures effectively from large databases and also the computational cost is high for the discovery. Probabilistic dependence is one technique, used to represent causality. Probabilistic cause–effect relationships have been examined and suggested in the literature (Reinchenbach 1978; Reichenbach and Reichenbach 1991; Good 1959; Suppes 1970). More recently, Bayesian networks (Pearl 2014), graphical causal modeling have emerged as a leading technique for discovering causal relationships. Authors (Heckerman 1995, 1997; Zhang and Poole 1996; Waldmann and Martignon 1998; Nadkarni and Shenoy 2001) describe the techniques they have proposed for characterizing, interpreting and learning probabilistic independence among parameters. However, Bayesian network learning to discover complete cause–effect models is an NP-complete problem (Chickering 1996). Constraint-based techniques are more efficient by avoiding the search for a generic Bayesian network. Currently, several constraint-based approaches have been implemented to identify causal relationships in large databases and achieved some satisfactory results (Cooper 1997; Silverstein et al. 2000; Mani et al. 2012; Pellet and Elisseeff 2008; Aliferis et al. 2010). These approaches use observational data to detect and learn causal structures using conditional independence among variables. It is significantly notable that these constraints-based approaches directly or indirectly implement the concept of Bayesian network learning, by creating a directed acyclic graph (DAG) which describes the conditional independence between variables (parameters). Even constraint-based methods shown promising results with large databases, they typically are designed to detect causality with few fixed structures in a directed acyclic graph (DAG), such as Y structures (Mani et al. 2012), CCC (Cooper 1997), and CCU (Silverstein et al. 2000).

Another technique in this area is Granger causality (GC) (Granger 1969). It has also been discussed in the previous literature (Lozano et al. 2009a, b; Arnold et al. 2007; Pang and Su 2010) and well known in economics causal inference. The method calculates the impact of one time series on another by finding out whether the response prediction can be improved by including the knowledge of a predictor or not. GC is reported to perform well for stationary time series data but is sensitive to non-linearity. All these methods infer directed networks. Although these methods are fast and, the inferred interactions are undirected. Moreover, these approaches are well suited for small sample data analysis (Veiga et al. 2007) but are not designed to detect combined causal parameters. Most of the time, two or more parameters may enhance the strength of effects. Even when individual parameter does not cause more effect, together they may do. We noticed that discovering causal structures in observational data only is insufficient. So, the discovered relationships have to be verified with time series data and controlled experiments. Still, it is acceptable to remove noncausal relationships discovered from data. Cause–effect relationship discovery is to find a brief list of rules that are probably causal. These causal rules provide a set of statistically decisive relationships which are acceptable to embed cause–effect relationships. This differentiates between the causal and normal rule discovery.

Association rule mining (Agrawal et al. 1993) has an efficient and versatile means for discovering relationships in data (Han et al. 2011). Authors (Jin et al. 2012; Li et al. 2013; Ma et al. 2016) use the advantage of association rule mining for causality discoveries. Jin et al. (2012) discovers the causal relationships with multiple cause variables in large databases of binary variables and excludes non-causal associations. Researchers (Li et al. 2013; Ma et al. 2016) discover potential causal rules using cohort study (Euser et al. 2009; Fleiss et al. 2003) and capable to generate combine causal rules in observational data. Author (Li et al. 2015) presented four approaches PC, HITON-PC, CR-PA and CR-CS for causality detection around a given target variable and discuss their efficiency. The PC and HITON-PC methods are based on Bayesian network learning theory and use conditional independence tests to eliminate non persistent associations, CR-PA use association rule and partial association and CR-CS uses the concept of a cohort study.

These proposed methods are able to find single and combined causal rules effectively in small and large database with low and high dimensional data, but they are restricted to discrete data and unable to extract the cyclic relationships and strength of relationships, although causality can be observed in various hidden relationships. However, statistically predictable associations do not illustrate cause–effect relationships, although mostly causality is usually observed as an association in the dataset. Therefore, in this paper, initially we use the concept of temporal association (Ji et al. 2011) and odds ratio (Fleiss et al. 2003) to extract binary causal relationship and further other relationships are extracted.

To the best of our knowledge, there is no previous work on discovering cyclic and transitive causal relationships with properties as the rate of change of parameters and their relationship strength in time series data. We should observe that discovering causal relationships in observational and constraint-based data only are insufficient.

The contributions of this work are listed in the following:

- First, we present a method to extract cause–effect relationships like binary, transitive, many to one and cyclic in large time series database.
- Second, we define the concept of temporal association lag rule and temporal odds ratio to extract cause–effect relationships between various parameters.
- Third, we are generating more specific cause–effect rules like binary, transitive, many to one and cyclic with their relationship strength which is useful for strategic decisions.

Our proposed method is useful to extract time lagged relationships across different field indicators that can be used to understand the lagged response of one indicator on another and various relationships such as binary, cyclic, many to one and transitive. We show the utility of our approach by extracting some relationships between different field indicators. For example, the rule (*Cereal production, D, 2* *%, 2*) $\Rightarrow$ (*Agricultural raw materials exports, 3* *%*), indicates a causal rule that cereal production is directly related to agricultural raw materials exports and if it is changed by 2 %, it affects the export of agricultural raw material by 3 % after 2 years. The proposed approach can be broadly applied to other problems in the temporal domain to extract various time lagged relationships.

## Preliminaries

In this section, first we define the terms used in this paper. Then we define the concepts for describing proposed cause–effect relationship extraction method. Finally, we describe the formal definition of various cause–effect relationships, discovering such causal relationships is the aim of this paper.

This paper deals with continuous parameters. Since all the parameters are having different ranges and we are interested in finding relationships. So instead of taking the absolute value of parameters, the rate of change is used to extract the effect of change of one parameter on another parameter, each time series value is categorized as a positive rate of change (*U*), a negative rate of change (*D*) and no rate of change (*Q*). To find an association between two parameters temporal association rule is used and defined using following terms:

*n*: Number of elements in time-series
*z*: Number of parameters in database *P*
*l*: Lag parameter, *l* ≠ 0
*l*_*max*_: Maximum lag difference value
*T*_*k*_: Value of *k*th time unit
*P*_*i,k*_: Value of *P*_*i*_ parameter in *k*th time unit
*γ*_*i,k*_: Rate of change of parameter *P*_*i*_ in *k*th year, can be calculated as:
  1 $$\gamma_{i,k} = \frac{{P_{i,k} - P_{i,k - 1} }}{{P_{i,k - 1} }}$$
$$\delta$$
Minimum rate of change used to consider a significant change
*R*_*i*,*k*_: Parameters indicate type of change, defined as:
  2 $$R_{i,k} = \left\{ {\begin{array}{*{20}l} {U\quad if \quad \, \gamma_{i,k} \ge \delta } \hfill \\ {D \quad if\quad \,\gamma_{i,k} \le - \delta } \hfill \\ {Q \quad if\quad - \delta \le \gamma_{i,k} \le \delta } \hfill \\ \end{array} } \right\}$$

The time series of parameter *P*_*i*_ is converted into a set of tuple 〈*P*_*i*_, *T*_*k*_, *R*_*i,k*_〉 where *T*_*k*_ is *k*th time period and *R*_*i*_ = *R*_*i,k*_ ∊ {*U*, *D*, *Q*} indicates the positive, negative or no rate of change for *k*th time unit. For example, if GDP is having a positive rate of change in 1970 than it is indicated by tuple 〈GDP, 1970, U〉.

Based on above structure of time series, the relationship between two parameters *P*_*i*_ and *P*_*j*_ for lag *l* is defined using following terms:

*D*_*i*,*j*,*k*,*l*_: Parameters indicate direct relationship, defined as:
  3 $$D_{i,j,k,l} = \left\{ {\begin{array}{*{20}l} 1 \hfill &\quad {if\,(R_{i,k} = U\,and\,R_{j,k + l} = U)\,or\,(R_{i,k} = D\,and\,R_{j,k + l} = D)} \hfill \\ 0 \hfill &\quad {otherwise} \hfill \\ \end{array} } \right\}$$
  i.e. the rate of change of *P*_*i*_ matches with the rate of change of *P*_*j*_ after time period *l*
$$S_{D} (P_{i} ,P_{j} ,l)$$
Support count of direct relationship, defined as:
  4 $$S_{D} (P_{i} ,P_{j} ,l) = \mathop \sum \limits_{k = 1}^{n - l} D_{i,j,k,l}$$
$$\alpha_{D} (P_{i} ,P_{j} ,l)$$
Support percent of direct relationship, defined as:
  5 $$\alpha_{D} (P_{i} ,P_{j} ,l) = \frac{{S_{D} (P_{i} ,P_{j} ,l)}}{n - l}$$
$$I_{i,j,k,l}$$
Parameters indicate inverse relationship, defined as:
  6 $$I_{i,j,k,l} = \left\{ {\begin{array}{*{20}l} 1 \hfill &\quad {if\,(R_{i,k} = U\, and\, R_{j,k + l} = D) \,or\, (R_{i,k} = D\, and\, R_{j,k + l} = U)} \hfill \\ 0 \hfill & \quad{otherwise} \hfill \\ \end{array} } \right\}$$
  i.e. the rate of change of *P*_*i*_ is opposite to rate of change of *P*_*j*_ after time period *l*
$$S_{I} (P_{i} ,P_{j} ,l)$$
Support count of inverse relationship, defined as:
  7 $$S_{I} (P_{i} ,P_{j} ,l) = \mathop \sum \limits_{k = 1}^{n - l} I_{i,j,k,l}$$
$$\alpha_{I} (P_{i} ,P_{j} ,l)$$
Support percent of inverse relationship, defined as:
  8 $$\alpha_{I} (P_{i} ,P_{j} ,l) = \frac{{S_{I} (P_{i} ,P_{j} ,l)}}{n - l}$$
$$\varTheta_{R}$$
Strength of relationship. It indicates toughness of relationship exists between parameters. The relationship between *P*_*i*_ and *P*_*j*_ is calculated as:
  9 $$\begin{aligned} \varTheta_{R} \left( { P_{i} , P_{j} } \right) & = \alpha * \log \left( n \right),\quad {\rm where} \quad \\ \alpha & = \alpha_{D} \left( {P_{i} ,P_{j} ,l} \right)\quad or\quad \alpha_{I} \left( {P_{i} ,P_{j} ,l} \right) \\ \end{aligned}$$

With our approach, we first consider the temporal association between indicators *P*_*i*_ and *P*_*j*_ since an association is needed for a cause–effect relationship. User defined support count threshold are defined as follows:

*α*_1_: Support count threshold for all causal relationships (considered as 70 % for experimentation).
*β*: Threshold for temporal odds ratio (considered as 3 for experimentation)

Since *α*_1_ is set 70 %, *β* is set to 3.

### **Definition 1**

(*Temporal association*) Using direct or indirect relationship [Eqs. (3)–(8)] temporal association can be defined as follows.

*Temporal direct association* Temporal direct association between two parameters *P*_*i*_ and *P*_*j*_ for time lag *l* is defined as $P_{i} \mathop \to \limits^{l} P_{j} \,if\, \alpha_{D} (P_{i} ,P_{j} ,l) \ge \alpha_{1}$.

*Temporal inverse association* Temporal inverse association between two parameters *P*_*i*_ and *P*_*j*_ for time lag *l* is defined as $P_{i} \mathop \to \limits^{l} P_{j} \,if\, \alpha_{I} (P_{i} ,P_{j} ,l) \ge \alpha_{1}$.

Next, we define the terms to calculate the temporal odds ratio of temporally associated parameters to check whether the temporal association rule $P_{i} \mathop \to \limits^{l} P_{j}$ is also causal rule or not.

$C_{E} (P_{i} ,P_{j} ,l)$ = Count of the number of pairs when no rate of change in *P*_*i*_ is associated with positive or negative rate of change in *P*_*j*_ after time period *l*, defined as:

10 $$C_{E} (P_{i} ,P_{j} ,l) = \mathop \sum \limits_{k = 1}^{n - l} E_{i,j,k,l}$$

where $E_{i,j,k,l}$ = Parameters indicate neutral-change relationship, defined as:

11 $$E_{i,j,k,l} = \left\{ {\begin{array}{*{20}l} 1 \hfill &\quad {\, if\,\,(R_{i,k} = Q\,\, and\,\,R_{j,k + l} = U)\,\,or\,\, \left( {R_{i,k} = Q\,\, and\,\,R_{j,k + l} = D} \right)} \hfill \\ 0 \hfill &\quad {otherwise} \hfill \\ \end{array} } \right\}$$

$C_{F} (P_{i} ,P_{j} ,l)$ = Count of the number of pairs when the positive or negative rate of change in *P*_*i*_ is associated with no rate of change in *P*_*j*_ after time period *l*, defined as:

12 $$C_{F} (P_{i} ,P_{j} ,l) = \mathop \sum \limits_{k = 1}^{n - l} F_{i,j,k,l}$$

where $F_{i,j,k,l}$ = Parameters indicate change-neutral relationship, defined as:

13 $$F_{i,j,k,l} = \left\{ {\begin{array}{*{20}l} 1 \hfill &\quad {if\,\,(R_{i,k} = U\,\,and\,\,R_{j,k + l} = Q) \,\,or\,\,(R_{i,k} = D\,\, and\,\,R_{j,k + l} = Q)} \hfill \\ 0 \hfill &\quad {otherwise} \hfill \\ \end{array} } \right\}$$

$C_{N} (P_{i} ,P_{j} ,l)$ = Count of the number of pairs when no rate of change in *P*_*i*_ is associated with no rate of change in *P*_*j*_ after time period *l*, defined as:

14 $$C_{N} \left( {P_{i} ,P_{j} ,l} \right) = \mathop \sum \limits_{k = 1}^{n - l} N_{i,j,k,l}$$

where $N_{i,j,k,l}$ = Parameters indicate neutral relationship, defined as:

15 $$N_{i,j,k,l} = \left\{ {\begin{array}{*{20}l} 1 \hfill &\quad { if\,\,(R_{i,k} = Q\,\,and\,\,R_{j,k + l} = Q)} \hfill \\ 0 \hfill &\quad {otherwise} \hfill \\ \end{array} } \right\}$$

### **Definition 2**

(*Temporal odds ratio*) It quantifies how strongly the presence or absence of change in value of parameter *P*_*i*_ effecting change in value of parameter *P*_*j*_. Using above terms [Eqs. (11)–(16)] temporal odds ratio is defined as follows.

*Temporal direct odds ratio* Temporal direct odds ratio between two parameters *P*_*i*_ and *P*_*j*_ for time lag *l* is defined as:

16 $$OR_{D} \left( {P_{i} ,P_{j} ,l} \right) = Oddratio_{D} \left( {P_{i} ,P_{j} ,l} \right) = \frac{{S_{D} \left( {P_{i} ,P_{j} ,l} \right)*C_{N} \left( {P_{i} ,P_{j} ,l} \right)}}{{C_{E} \left( {P_{i} ,P_{j} ,l} \right)* C_{F} \left( {P_{i} ,P_{j} ,l} \right)}}$$

*Temporal inverse odds ratio* Temporal inverse odds ratio between two parameters *P*_*i*_ and *P*_*j*_ for time lag *l* is defined as:

17 $$OR_{I} \left( {P_{i} ,P_{j} ,l} \right) = Oddratio_{I} \left( {P_{i} ,P_{j} ,l} \right) = \frac{{S_{I} \left( {P_{i} ,P_{j} ,l} \right)}}{{C_{E} \left( {P_{i} ,P_{j} ,l} \right)* C_{F} \left( {P_{i} ,P_{j} ,l} \right)}}$$

In our experimentation, if the value of $C_{N} (P_{i} ,P_{j} ,l)\,\, or\,\, C_{E} (P_{i} ,P_{j} ,l) \,\,or \,\,C_{F} (P_{i} ,P_{j} ,l)$ between parameters is zero, we considered it as 1 to avoid infinite temporal odds ratio.

Further causal rules are defined using terms define in Definitions 1 and 2.

### **Definition 3**

(*Binary rule*) A binary causal rule $(P_{i} , D, l) \Rightarrow (P_{j} )$, exists between *P*_*i*_ and *P*_*j*_ if there is temporal association rule $P_{i} \mathop \to \limits^{l} P_{j} \,{\text{and}}\, Oddratio_{D} (P_{i} ,P_{j} ,l) \ge \beta \,{\text{or}}\,Oddratio_{I} (P_{i} ,P_{j} ,l) \ge \beta$.

In experimentation results, we represent direct causal rule by $(P_{i} , D, l) \Rightarrow (P_{j} )$ and inverse by $(P_{i} , I, l) \Rightarrow (P_{j} )$.

This rule will serve as a forward pruning criterion where all parameters which are not associated with another parameter with non-zero lag value are excluded from the combination of future search. The minimum required support makes the search space manageable.

### **Definition 4**

(*Precise binary rule*) A precise binary rule $(P_{i} , D, \delta_{1} ,l) \Rightarrow (P_{j} ,\delta_{2} )$, exists between *P*_*i*_ and *P*_*j*_ if there is binary rule $(P_{i} , D, l) \Rightarrow (P_{j} )$ and $\left( {\delta = \delta_{1} } \right),$ i.e. minimum growth rate of change of *P*_*i*_ and $\left( {\delta = \delta_{2} } \right),$ i.e. minimum growth rate of change of *P*_*j*_ and the rule will not hold either $\delta > \delta_{1} \,\,for \,\,P_{i } \,\, or\,\, \delta > \delta_{2} \,\,for\,\,P_{j}$.

### **Definition 5**

($fscore (\delta_{1} ,\delta_{2} )$) A function is used to calculate the specificity of the rule. In the experimentation, it is defined as $fscore (\delta_{1} ,\delta_{2} ) = \delta_{1}^{2} + \delta_{2}^{2}$. If rule $(P_{i} , D, \delta_{1} ,l_{1} ) \Rightarrow (P_{j} ,\delta_{2} )$ is satisfied for multiple value of $\delta_{1} ,\delta_{2}$ than the rule which gives the maximum valid *fscore* is retained.

Based on binary causal rule, we try to extract other causal relationships as transitive, many to one (combined cause) and cyclic. We define these relationships as follows.

### **Definition 6**

(*Transitive rule*) A transitive rule $$(P_{i} , D, \delta_{1} ,l_{1} ) \Rightarrow (P_{j} ,D,\delta_{2} ,l_{2} ) \Rightarrow \left( {P_{k} , \delta_{3} } \right),$$ exists between *P*_*i*_, *P*_*j*_ and *P*_*k*_ if there is $$r1:(P_{i} , D, \delta_{1} ,l_{1} ) \Rightarrow (P_{j} ,\delta_{2} ), r2: (P_{j} , D, \delta_{2} ,l_{2} ) \Rightarrow (P_{k} ,\delta_{3} ), (P_{i} , D, \delta_{1} ,l_{3} ) \Rightarrow (P_{k} ,\delta_{3} ),$$$$l_{3} \ge l_{1} + l_{2} \,and\, r_{1} (P_{j} ) \cap r_{2} (P_{j} ) \ne \emptyset$$.

### **Definition 7**

(*Combined cause rule*) A many to one rule $$\left( {\left( {P_{i} , D, \delta_{1} ,l_{1} } \right),\left( {P_{j} ,D,\delta_{2} ,l_{2} } \right)} \right) \Rightarrow (P_{k} ,\delta_{3} ),$$ exists between *P*_*i*_, *P*_*j*_ and *P*_*k*_ if there is $$(P_{i} , D, \delta_{1} ,l_{1} ) \Rightarrow (P_{k} ,\delta_{3} ), (P_{j} , D, \delta_{2} ,l_{2} ) \Rightarrow (P_{k} ,\delta_{3} ), S_{D} \left( {P_{i} ,P_{k} ,l_{1} } \right) \ge \alpha_{1} ,$$$$S_{D} (P_{j} ,P_{k} ,l_{2} ) \ge \alpha_{1} \, and\,S_{D} ((P_{i} , P_{j} ),P_{k} ,l_{1} ,l_{2} ) \ge \alpha_{1}$$.

### **Definition 8**

(*Cyclic rule*) A cyclic rule $(P_{i} , D, \delta_{1} ,l_{1} ) \Leftrightarrow (P_{j} ,D,\delta_{2} ,l_{2} ),$ exists between *P*_*i*_ and *P*_*j*_ if there is $(P_{i} , D, \delta_{1} ,l_{1} ) \Rightarrow (P_{j} ,\delta_{2} ), (P_{j} , D, \delta_{2} ,l_{2} ) \Rightarrow (P_{i} ,\delta_{1} ) \,\,and\,\,S_{D} ((P_{i} , P_{j} ),l_{1} ,l_{2} ) \ge \alpha_{1}$.

## Proposed method

In this section, we described an algorithm based on the definitions. The algorithm is explained in five steps. Step 1 generates the binary causal rule. Step 2 generates more precise rules of binary causal rules. Steps 3, 4, and 5 generate the transitive, many to one and cyclic rules. Further, we give the explanation of each step of an algorithm. Table 1 represents the abbreviations used in the algorithm and in this paper. Let *P* be a time series database in discrete form and *P*_*i*_ is a time series of parameter *P*_*i*_ have *U*, *D*, and *Q* values as mentioned in the definitions, *z* is a number of parameters in database *P*.

**Table 1:** Abbreviation table

Abbreviation	Description
TOR	Temporal odds ratio
BRS	Binary rule set
SRS	Specific rule set
TRS	Transitive rule set
MOS	Many to one rule set
CRS	Cyclic rule set
AG	Agriculture land
AR	Arable land
ARME	Agricultural raw materials exports
CAB	Current account balance
CY	Cereal yield
CO2	CO2 emissions
CP	Crop production
CPI	Crop production index
EDOE	Electronic data processing and office equipment
FDI	Foreign direct investment
FMP	Fuels and mining products
FR	Forest rents
GDP	Gross domestic product
GGR	General government revenue
GNS	Gross national savings
I_1_ to I_10_	No of indicators (10)
ICEC	Integrated circuits and electronic components
IS	Iron and steel
OM	Other manufactures
OTE	Office and telecom equipment
TI	Total investment
VEG	Volume of exports of goods
VEGS	Volume of exports of goods and services
VIG	Volume of imports of goods

### Step 1: Binary rule generation


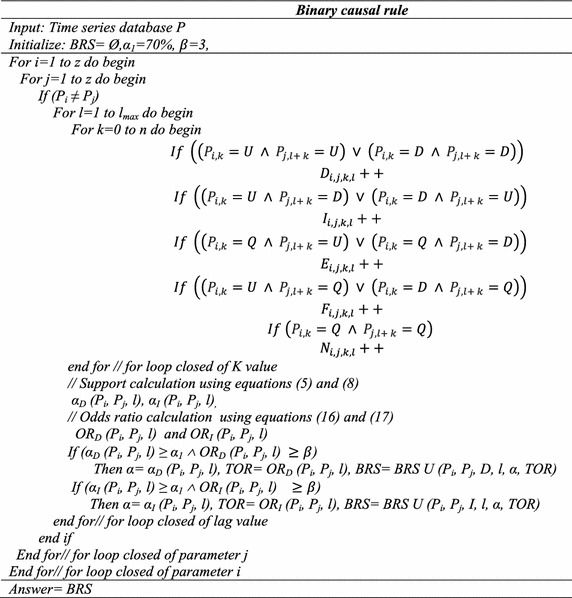


A causal rule may be generated for multiple lag values, the lag value which gives maximum support of rule will be considered. Suppose *P* = {*P*_1_*, P*_2_, *P*_3_, *P*_4_, *P*_5_}, set of time series dataset and using this step 1 BRS generated results are as follows.

BRS = {(*P*_1_, *P*_2_, *D*, l, 75, 4), (*P*_1_*, P*_3_*, D*, 2, 73, 4), (*P*_2_*, P*_3_*, I*, l, 77, 3), (*P*_4_*, P*_3_*, I,* l, 71, 6), (*P*_2_*, P*_5_*, D*, l, 76, 5), (*P*_5_*, P*_2_*, D*, l, 72, 4)}. Here (*P*_1_*, P*_2_*, D*, l, 75, 4), describes that parameters *P*_1_ and *P*_2_ have a direct relationship with lag 1, support 75 and TOR = 3, which indicates that (*P*_1_*, P*_2_) are causally related, i.e. *P*_1_ effects *P*_2_ after 1 year. Similarly, by comparing support and their odds ratio between parameters for each tuple, the other binary causal relationship can be extracted and interpreted.

#### *Explanation*

To describe this step, we consider the time series using rate of change as positive (*U*) or negative (*D*) of two parameters say *P*_*i*_ and *P*_*j*_ for a time period (91–97).

Let

- *T* = {1991, 1992, 1993, 1994, 1995, 1996, 1997}
- *P*_*i*_ = {*U, U, U, U, U, D, U*}
- *P*_*j*_ = {*D, U, U, U, U, U, U*}

Here we calculate support value α for lag value = 1.

Support value for lag value 1 *α*_*D*_ (*P*_*i*_*, P*_*j*_, 1) = 83 % and temporal odd ratio (TOR), *Oddratio*_*D*_ (*P*_*i*_*, P*_*j*_, 1) = 5.

Since calculated *α*_*D*_ > *α*_1_ and TOR > 3 the rule $(P_{i} , D, 1) \Rightarrow (P_{j} )$, is correct and exists for lag value 1 (i.e. l ≠ 0).

Relationship strength [using Eq. (10)] of this rule is, 70.13.

If time series data are given for some parameters, we can calculate *α*_*D*_ and TOR between parameters and rules can be extracted. So with the help of the above algorithm, we would be able to extract all two-variable causal relationships between parameters for a time series data set.

### Step 2: Specific rules generation

In this step, we calculated the specific rule for binary causal rules generated in the above algorithm.

Let $\gamma_{i}$ and $\gamma_{j}$ are the rate of change of parameters *P*_*i*_ and *P*_*j*_ and parameters have a direct relationship.

$Let \delta_{i} max$ = maximum value of the rate of change of *P*_*i*_, $\delta_{j} max$ = maximum value of the rate of change of *P*_*j*_, $\delta_{i} min$ = minimum value of the rate of change *P*_*i*_, $\delta_{j} min$ = minimum value of the rate of change *P*_*j*_.

Calculation of interval value $\upeta_{{P_{i} }}$ (increment, value for a parameter *P*_*i*_)

18 $$\eta_{{P_{i} }} = \frac{{\delta_{i} \hbox{max} - \delta_{i} \hbox{min} }}{n} \,\,\,and\,\,\,\eta_{{P_{j} }} = \frac{{\delta_{j} \hbox{max} - \delta_{j} \hbox{min} }}{n}$$

where $$\delta_{i} \hbox{max} \,\,or\,\,\delta_{j} \hbox{max} = \mu + 2\sigma \,\,and\,\,\delta_{i} \hbox{min} \,\,or\,\,\delta_{j} \hbox{min} = \mu - 2\sigma$$
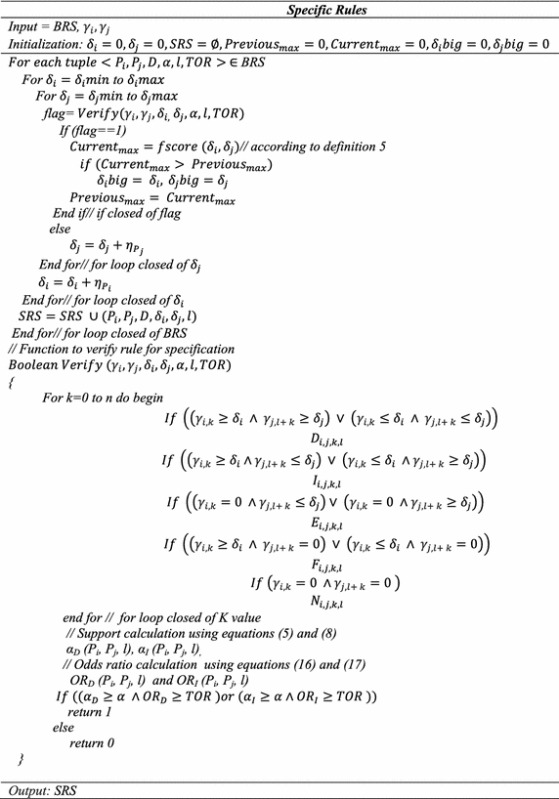


Let $\delta_{1} , \delta_{2}$ is the minimum rate of change of parameters *P*_*i*_*, P*_*j*_. Then, using this step 2 more specific causal rules $(P_{i} , D, \delta_{1} ,l) \Rightarrow (P_{j} ,\delta_{2} )$ can be generated. The rule indicates that *P*_*i*_ and *P*_*j*_ have a direct causal relationship with lag 1 and if *P*_*i*_ is changed by $\delta_{1}$ it leads to change *P*_*j*_ by $\delta_{2}$. Based on BRS results assumed in step 1 more specific rules can be generated as follows:

SRS = {(*P*_1_*, P*_2_*, D*, 1 %, 2 %, 1), (*P*_1_*, P*_3_*, D*, 2 %, 1 %, 2), (*P*_2_*, P*_3_*, D*, 2 %, 1.5 %, 1), (*P*_4_*, P*_3_*, I*, 1.5 %, 2 %, l), (*P*_2_*, P*_5_*, D*, 2 %, 3 %, 1), (*P*_5_*, P*_2_*, I*, 3 %, 2 %, 1)}.

### Step 3: Transitive rule generation


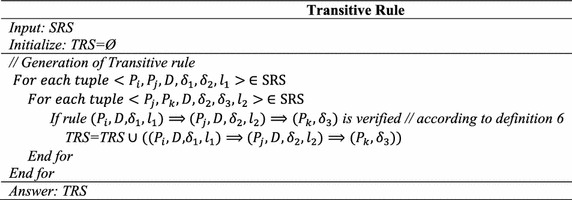


Based on SRS results in step 2, tuple (*P*_1_*, P*_2_*, D*, 1 %, 2 %, 1), (*P*_2_*, P*_3_*, D*, 2 %, 1.5 %, 1) and (*P*_1_*, P*_3_*, D*, 2 %, 1 %, 2) satisfies all the conditions of transitive relation and generate a transitive rule

$$(P_{1} , D, 1\% ,1) \Rightarrow (P_{2} ,D, 2\% ,1) \Rightarrow \left( {P_{3} , 1\% } \right)$$

If the same parameter has a different rate of change in different rules minimum of them is considered.

#### *Explanation*

To understand this, we consider the time series of three parameters *P*_*i*_*, P*_*j*_, and *P*_*k*_ as follows.

Let TOR > 3 and $\delta_{1} , \delta_{2} , \delta_{3}$ is the rate of change of parameters $P_{i} , P_{j} , P_{k} .$ Calculate support values from Table 2 is:

- $${\text{Support}}\,{\text{value}}\,{\text{of}}\,P_{i} \left( U \right)\,{\text{and}}\,P_{j} \left( D \right),\,\alpha_{ij} \left( {P_{i} , P_{j} ,1} \right) = 77.7.$$
- $${\text{Support}}\,{\text{value}}\,{\text{of}}\,P_{j} \left( D \right)\,{\text{and }}\,P_{k} \left( D \right),\alpha_{jk} \left( {P_{j} , P_{k} ,1} \right) = 88.8,$$
- $${\text{Support}}\,{\text{value}}\,{\text{of}}\,P_{i} \left( D \right)\,{\text{and }}\,P_{k} \left( D \right),\alpha_{ik} \left( {P_{i} , P_{k} ,2} \right) = 75,$$

Since $\alpha_{ij} > \alpha_{1} , \alpha_{jk} > \alpha_{1} , \alpha_{ik} > \alpha_{1} ,$ generated binary causal rules are

$$(P_{i} , I, \delta_{1} ,1) \Rightarrow (P_{j} ,\delta_{2} ), (P_{j} , D, \delta_{2} ,1) \Rightarrow (P_{k} ,\delta_{3} ), (P_{i} , I, \delta_{1} ,2) \Rightarrow (P_{k} ,\delta_{3} ).$$

The condition *l*_3_ ≥ 2 (1 + 1) is also satisfies and generated transitive rule is $(P_{i} , I, \delta_{1} ,1) \Rightarrow (P_{j} ,D,\delta_{2} ,1) \Rightarrow (P_{k} , \delta_{3} )$.

**Table 2:** Parameter time series

Time	P_i_	P_j_	P_k_
1991	U	D	U
1992	U	D	D
1993	U	D	D
1994	U	D	D
1995	U	D	D
1996	D	D	D
1997	D	D	D
1998	U	D	U
1999	D	D	D
2000	U	D	D

### Step 4: Many to one (combined causal) rule generation


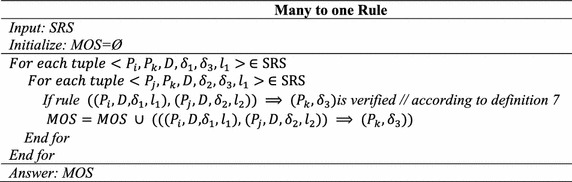


Based on SRS results in step 2, tuple (*P*_1_*, P*_3_*, D*, 2 %, 1 %, 2), (*P*_4_*, P*_3_*, I*, 1.5 %, 2 %, l) and using this step 4 generated combined causal rule is $((P_{1} , D, 2\% ,2),(P_{4} ,I,1.5\% ,1)) \Rightarrow (P_{3} ,1\% ).$

#### *Explanation*

 Let we have the following values for parameters $P_{i} , P_{j} , {\text{and }}P_{k}$.

 Let TOR > 3, $\delta_{1} , \delta_{2} , \delta_{3}$ is the rate of change of parameters $P_{i} , P_{j} , P_{k}$. Calculate support values from Table 3 as: ${\text{Support}}\,{\text{value}}\,{\text{of}}\,\alpha_{ik} \left( {P_{i} , P_{k} ,1} \right) = 77.7\,\% ,\, {\text{Support}}\,{\text{value }}\,{\text{of}}\, \alpha_{jk} \left( {P_{j} , P_{k} ,1} \right) = 88.8\%$.

**Table 3:** Parameter time series

**Time**	P_i_	P_j_	P_k_
1991	*U*	*U*	D
1992	*U*	*U*	*D*
1993	U	D	*D*
1994	D	U	D
1995	*U*	*U*	D
1996	*U*	*U*	*D*
1997	*U*	*U*	*D*
1998	*U*	*U*	*D*
1999	*U*	*U*	*D*
2000	U	U	*D*

Italic letters indicate the temporal association between parameters for given time. For example, P_i_ and P_j _are associated for lag 0 in 1991 and (P_i_, P_j_ ) are associated with P_k_ at lag 1. So, P_i_ and P_j_ values are italic at 1991 and P_k_ at 1992

Calculated support values $\alpha_{ik} , \alpha_{jk} \,{\text{and}}\,\alpha_{ijk} > \alpha_{1}$ which satisfies Definitions 4 and 7. In Table 3 highlighted rows indicates the $((P_{i} , P_{j} ), P_{k} )$ relationship. Since all the conditions are satisfied the generated combined rule is$((P_{i} ,I,\delta_{1} ,1),(P_{j} ,I,\delta_{2} ,1)) \Rightarrow (P_{k} , \delta_{3} )$.

### Step 5: Cyclic rule generation


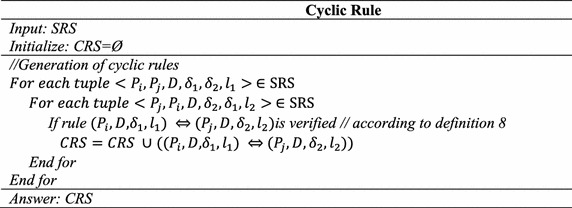


Based on SRS results in step 2, tuple (*P*_2_*, P*_5_*, D*, 2 %, 3 %, 1), (*P*_5_*, P*_2_*, I*, 3 %, 2 %, 1) and using this step generated cyclic rule is $(P_{2} , D, 2\% ,1) \Leftrightarrow \left( {P_{5} , D,3\% , 1} \right)$.

#### *Explanation*

To understand this rule, we consider two parameters say *P*_*i*_, and *P*_*j*_, for a time period 1998–2015. Let $\delta_{1} \, {\text{and}}\, \delta_{2}$ are rate of change for parameters $P_{i} , P_{j}$ which have the following values.

We can identify that relationship $(P_{i} , I, \delta_{1} ,1) \Rightarrow (P_{j} ,\delta_{2} )$, $(P_{j} , I, \delta_{2} ,1) \Rightarrow (P_{i} ,\delta_{1} ),$ are satisfied in Table 4 from Definition 4. In Table 4, the time period satisfies cyclic relation between parameters is {(1988–1991), (1990–1992), (1992–1994), (1993–1995), (1995–1997), (1996–1998)}. For example (1988–1991) indicates that if *P*_*i*_ increases in 1988 *P*_*j*_ goes down in 1989 which in turn increases *P*_*i*_ in 1990.

**Table 4:** Parameter time series

Time	P_i_	P_j_
1988	U	U
1990	U	D
1991	U	D
1992	U	D
1993	D	D
1994	U	U
1995	D	D
1996	D	U
1997	D	U
1998	D	U

Calculated support value *α*_*ij*_ for parameters *P*_*i*_ and *P*_*j*_: 75 %. Since *α*_*ij*_ > *α*_1_ cyclic relation is satisfied and generated cyclic causal rule is (*P*_*i*_, *I*, *δ*_1_, 1) ⇔ (*P*_*j*_, *I*, *δ*_2_, 1).

## Experiments

We implemented our method using Java programming language with Net Beans IDE 7.3. The computation time to check the causal relationship between parameters is high using serialized programming. So we use a parallelization approach in our program using threads in Java on a machine with configuration Dual-Core CPU contains 12-Cores, 8 GB RAM, and 64-bit Windows 7 Operating System. Our goal is to discover various causal relationships between the different economic parameters. Firstly, we find all the binary causal rules (i.e. one cause and one effect parameter) and then other causality rules are discovered using proposed method. For experimentation, minimum support threshold *α*_1_ is set 70 % and $\beta$ is set 3.

### Dataset

The approach is discussed using 2 synthetic and 3 real-world dataset. Table 5 shows the summary of data sets. The synthetic dataset is generated using R software based on Bayesian network (BN). First, we create random numbers, next build a BN on it and then generate the data from BN. Real world economic datasets are obtained from the World Trade Organization (1995), International Monetary Fund (1945) and World Bank data (1944). The WTO provides data on international trade in merchandise and commercial services. IMF contains time series data of 189 countries on economic parameters. World Bank contains time series data from 250 countries on a variety of topics such as agriculture, education, health, and an environment, etc. In World Bank and IMF, both we tested our algorithm for south-Asian countries (India, Pakistan, Sri Lanka, Bangladesh, Nepal, Bhutan and the Maldives, Afghanistan). In WTO, we used the data of Merchandise trade: Network of world merchandise trade in Asia.

**Table 5:** Datasets

Name	Length of time series (years)	No of indicators (parameters)
Synthetic-1	40	6
Synthetic-2	40	10
WTO	31	30
IMF	34	40
World Bank	52	1346

All the datasets are selected to test the effectiveness of proposed method. In our experiments first, we preprocess the continuous data set [Eq. (1)] and represented them by positive, negative and neutral (no) rate of change as *U*, *D,* and *Q* value [Eq. (2)] from the primitive data sets.

### Results

This section presents the various extracted causal relationships for World Bank data sets. Results on other datasets are shown in “Comparison” section. To save space, at below, we omitted all relationships and consider only those relationships which are present in multiple countries and displaying some of them. The discovered causal rules with our approach are shown in Table 6 for south-Asian countries. In Table 6 causal relationship between parameters is described with its support, strength and rate of change of indicators. For example, a rule (*Cereal production, D, 3* *%, 1)* ⇒ *(Crop production index, 1* *%*), indicates direct relationship, i.e. increase in cereal production by 3 %, will increase the crop production index by 1 % after 1 year. This rule is discovered in four countries Srilanka, Nepal, Pakistan and India with different strength and support values. On the basis of support and strength value, we can say that this rule is more valid for Nepal rather than the other three countries. We can also identify a rule which has more valid for a country. In Table 6 from the binary causal rule, we can observe that three rules are present in India and above discussed rule is more valid than other rules in India. The transitive causal rules: (*Rural population, D, 1* *%, 1*) ⇒ *Population density, D, 0.33* *%, 1*) ⇒ *Population, total, 0.68* *%*) can be described as, a 1 % increase in rural population increase population density by 0.33 % after 1 year, which tends to increase the total population by 0.68 % after a year. This rule is present in four countries, Afghanistan, India, Maldives, and Nepal. The rule is having more impact on India. As compared to binary and transitive causal rules, the algorithm extracts the less number of causal rules for many to one (combined causal) and cyclic. The many to one causal rule: {(*Forest rents, I, 5* *%, 2*), (*Foreign direct investment, D, 3* *%, 1*)} ⇒ *(Crop production index, 7* *%*) indicates that the decrease in forest rent by 5 % and increase in foreign direct investment by 3 % would tend to increase the crop production index by 7 %. The cyclic causal rule: (*Gross domestic savings, D, 1* *%, 1)* ⇔ (*Cereal yield, D, 0.5* *%, 2*) can be described as, a 1 % increase in gross domestic savings increase cereal yield by 0.5 % after a year and increases in cereal yield would again increase gross domestic savings after 2 years. Similarly, other rules in all causal relationships can be analyzed.

**Table 6:** Causality rules

Rules	Countries	Support	Strength
*Binary causal rules*			
(Cereal production, D, 2 %, 2) $\Rightarrow$ (agricultural raw materials exports, 3 %)	India	74	120.8767
	Pakistan	76	124.1436
(Air transport, D, 1 %, 2) $\Rightarrow$ (GDP growth, 0.22 %)	India	74	120.8767
	Nepal	79	129.0440
(Cereal production, D, 3 %, 1) $\Rightarrow$ (crop production index, 1 %)	Srilanka	76	124.1436
	Nepal	81	132.3109
	Afganistan	76	124.1436
	India	76	124.1436
*Transitive causal rules*			
(Rural population, D, 1 %, 1) $\Rightarrow$ (population density, D, 0.33 %, 1) $\Rightarrow$ (population total, 0.68 %)	Afghanistan	74	120.8767
	India	83	135.5779
	Maldives	77	125.7771
	Nepal	71	115.9763
(Land under cereal production, D, 3 %, 1) $\Rightarrow$ (food exports, D, 1 %, 2) $\Rightarrow$ (GDP growth, 1.5 %)	India	71	115.9763
	Pakistan	72	117.6097
	Bangladesh	71	115.9763
(Arable land, D, 1 %, 1) $\Rightarrow$ (agricultural land, D, 1 %, 3) $\Rightarrow$ (CO2 emissions, 1.5 %)	India	71	115.9763
	Srilanka	71	115.9763
	India	70	114.3428
*Many to one (combined causal) causal rule*			
{(Rural population, D, 2.3 %, 1), (urban population D, 0.5 %, 1)} $\Rightarrow$ (population density, 1 %)	India	79	129.0440
	Afghanistan	72	117.6097
	Pakistan	72	117.6097
{(Forest rents, I, 5 %, 2), (Foreign direct investment, D, 3 %, 1)} $\Rightarrow$ (crop production index, 7 %)	Srilanka	72	117.6097
{(Land under cereal production, D, 0.8 %, 1), (rural population, I, 1 %, 2)} $\Rightarrow$ (cereal production, 2 %)	Afghanistan	73	119.2432
	India	72	117.6097
	Pakistan	70	114.3428
*Cyclic causal rules*			
(Land under cereal production, D, 2.5 %, 2) ⇔ (agricultural land, D, 4.5 %, 1)	India	72	117.6097
(Gross domestic savings, D, 1 %, 1) ⇔ (cereal yield, D, 0.5 %, 2)	Srilanka	70	114.3428
	India	70	114.3428

### Prediction effectiveness

The rules can be validated by calculating the mutual information (Meyer 2014) between indicators and the conditional entropy (Marsh 2013; Meyer 2014) change of the indicator before and after applying the rule. It is shown in Table 7 that the indicators are mutually related and the entropy of the indicator is decreased after applying the rule.

**Table 7:** Entropy of indicators

Indicators	Target indicator entropy	Proposed method conditional entropy after applying rule	Mutual information between indicators
CP → ARME	1.0973	0.51	0.837
AG → AR → CO2	1.0986	0.58	0.585
(FDI, FR) → CPI	1.0972	0.035	0.595
GDP ←→ CY	1.0961	0.37	0.583

Table 7 results show that the target indicator entropy is decreased after the rule is applied, which represents that indicator value is more uncertain when it is considered alone. For example, the large value of mutual information between CP and ARME, indicates that the two indicators are related and the entropy of ARME is decreased after the rule *CP* → *ARME* is applied. So it can be concluded that the proposed method achieves high prediction effectiveness. We validated all the generated causal rules using the concept of decrease in entropy and mutual information to check their prediction effectiveness. Generated causal rules can also be validated using time series graphs shown in “Appendix”.

### Scalability

**Fig. 2:**
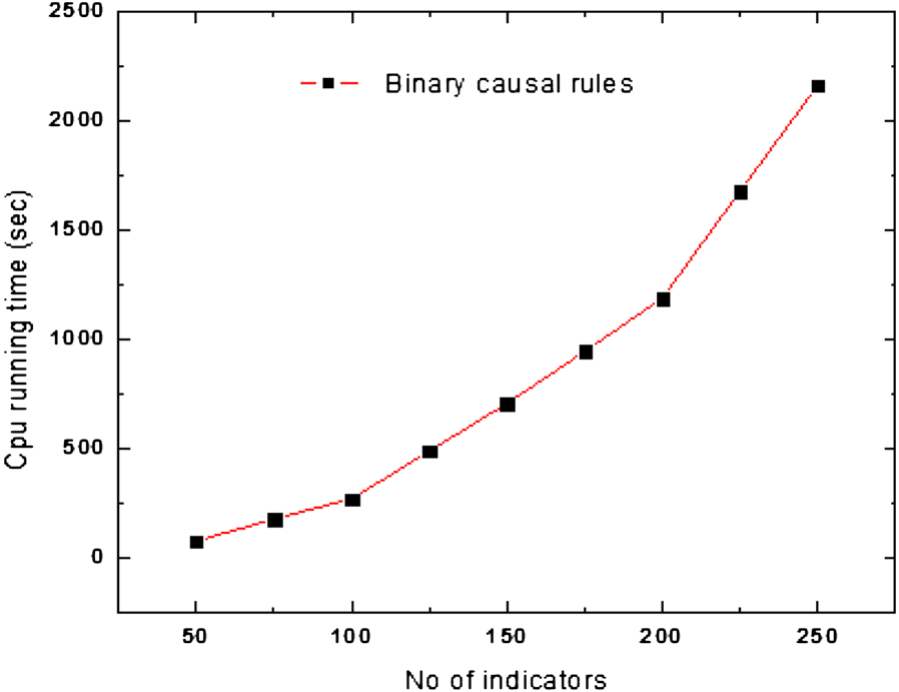
Scale up of indicators for binary causal rules

**Fig. 3:**
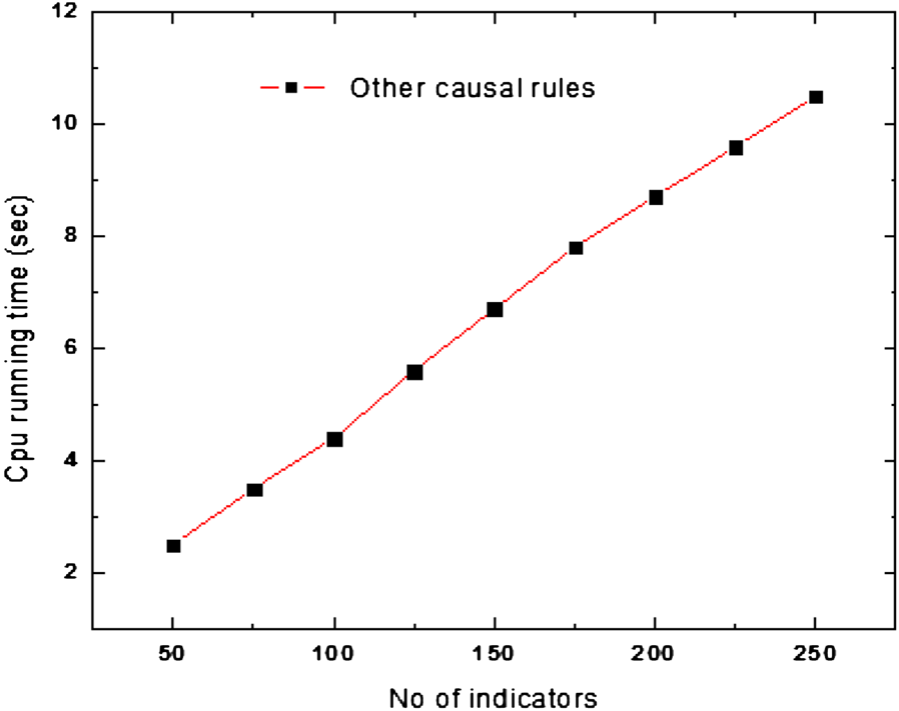
Scale up of indicators for other causal rules

Further, we do experimentation to evaluate the scalability of the algorithm with the involved years and the number of indicators. Considering Figs. 2 and 3, it could be seen that, the proposed cause–effect discovery method scales up with the number of indicators. We examine the performance degradation of the algorithm on the basis of various causal rule discoveries for nine different scales (number of indicators): 50, 75, 100, 125, 150, 175, 200, 225 and 250. The minimum support threshold is set 70, and it remains the same in all the experiments.

As shown in Fig. 2, the extraction time increases squarely with the number of indicators. More important, the curve is parabolic, which means that the performance of our algorithm is non-linearly related to the increase of number of indicators in binary causal rules. Though the time for generation of the binary causal rule is increasing squarely with a number of indicators, time for generation of other rules is not non-linear because the generation of other rules uses the result of binary rule generation (in Fig. 3).

The proposed method is able to extract nonlinear relationship from extracted causal rules because we are dealing with change of values as the rate of change and this change can be linear or nonlinear.

## Discussion

### Comparison

To assess the efficiency of the proposed method, we compared proposed method with both statistical and non statistical methods. Statistical (Granger causality, Bayesian network) methods comparison is performed using R software packages as lmtest (Hothorn et al. 2015) for GC and bnlearn (Scutar 2016) for BN. In BN we calculate the results using constraint based local discovery algorithm hiton.pc (Aliferis et al. 2003). For non-statistical approaches, we implemented the methods (Silverstein et al. 2000; Jin et al. 2012; Li et al. 2013) in Java for causal rule discovery.

First, we compared proposed method with GC and BN. GC is the base method to detect lag relationship in stationary time series data set. We run GC for different lag values with significance level, α = 0.05. HITON-PC is an effective algorithm of BN to extract parent–child relationship. So we considered both statistical methods as a benchmark for accuracy comparison. Tables 8 and 9 describe that all the binary rules which are generated in all the datasets by other methods are also generated by the proposed method. For example in the synthetic-2 dataset, we described the rule related to indicator I_7_ and I_8_. In the statistical approach from Table 8, we can observe that the GC can discover only binary causal rules while BN can discover transitive as well as binary rules between indicators. For example, in a BN graph like *I*_1_ → *I*_3_ → *I*_6_ can be generated, but *I*_1_ and *I*_6_ are independent, i.e. *I*_1_ and *I*_6_ may or may not be dependent. In proposed method *I*_1_ and *I*_6_ are conditionally dependent or *I*_1_ is an indirect cause of *I*_6_.

**Table 8:** Comparison of proposed method with statistical method

Dataset	Indicators relationships	Extracted rules	Statistical methods		
			Proposed method	Granger causality	Bayesian network
Synthetic-1 (I_1_–I_6_)	Binary	I_1_ → I_3_	✓	✓	✓
	Many to one	(I_2_, I_4_) → I_5_	✓		
	Transitive	I_1_ → I_3_ → I_6_	✓		✓
	Cyclic	I_1_ ←→ I_3_	✓		
Synthetic-2 (I_1_–I_10_)	Binary	I_1_ → I_7_, I_2_ → I_7_, I_7_ → I_2_, I_1_ → I_3_, I_7_ → I_8_	✓	✓	✓
	Many to one	(I_6_, I_9_) → I_7_	✓		
	Transitive	I_1_ → I_7_ → I_8_	✓		✓
	Cyclic	I_2_ ←→ I_7_	✓		
WTO	Binary	Chemicals → Textiles Chemicals → OTE	✓	✓	✓
	Many to one	(OTE, Textiles) → EDOE	✓		
	Transitive	IS → OM → ICEC	✓		✓
	Cyclic	OM ←→ IS	✓		
IMF	Binary	GGR → VEG	✓	✓	✓
	Many to one	(GGR, GNS) → TI	✓		
	Transitive	GDP → VIG → TI	✓		✓
	Cyclic	CAB ←→ VEGS	✓		
World Bank data	Binary	CP → ARME	✓	✓	✓
	Many to one	(FDI, FR) → CPI	✓		
	Transitive	AR → AG → CO2	✓		✓
	Cyclic	GDP ←→ CY	✓

**Table 9:** Comparison of proposed method with non statistical method

Dataset	Indicators relationships	Extracted rules	Non-statistical methods			
			Proposed method	Silverstein et al. (2000)	Jin et al. (2012)	Li et al. (2013)
Synthetic-1 (I_1_–I_6_)	Binary	I_1_ → I_3_	✓	✓	✓	✓
	Many to one	(I_2_, I_4_) → I_5_	✓		✓	✓
	Transitive	I_1_ → I_3_ → I_6_	✓	✓		
	Cyclic	I_1_ ←→ I_3_	✓			
Synthetic-2 (I_1_–I_10_)	Binary	I_1_ → I_7_, I_2_ → I_7_, I_7_ → I_2_, I_1_ → I_3_, I_7_ → I_8_	✓	✓	✓	✓
	Many to one	(I_6_, I_9_) → I_7_	✓		✓	✓
	Transitive	I_1_ → I_7_ → I_8_	✓	✓		
	Cyclic	I_2_ ←→ I_7_	✓			
WTO	Binary	Chemicals → Textiles Chemicals → OTE	✓	✓	✓	✓
	Many to one	(OTE, Textiles) → EDOE	✓		✓	✓
	Transitive	IS → OM → ICEC	✓	✓		
	Cyclic	OM ←→ IS	✓			
IMF	Binary	GGR → VEG	✓	✓	✓	✓
	Many to one	(GGR, GNS) → TI	✓		✓	✓
	Transitive	GDP → VIG → TI	✓	✓		
	Cyclic	CAB ←→ VEGS	✓			
World Bank data	Binary	CP → ARME	✓	✓	✓	✓
	Many to one	(FDI, FR) → CPI			✓	✓
	Transitive	AR → AG → CO2	✓	✓		
	Cyclic	GDP ←→ CY	✓			

Second, we compared our method with non-statistical methods. From Table 9 it can observe that binary and combined (many to one) causal relationship can be discovered by Jin et al. (2012) and Li et al. (2013) in all datasets. Silverstein et al. (2000) can also detect many to one rule but independently. For example, if we consider the rule (*I*_2_*, I*_4_) → *I*_5_ in the synthetic-1 dataset it would be considered as *I*_2_ → *I*_5_ ← *I*_4_, i.e. *I*_2_ and *I*_4_ affect *I*_5_ independently, so we have not considered the many to one rule generated in a method (Silverstein et al. 2000). A transitive relationship is extracted by Silverstein et al. (2000) and proposed method. Relationships extracted by various methods are shown in Tables 8 and 9.

Based on the experimental results, it is reasonable to conclude that proposed method is capable to extract various causal relationships and causal rules like cyclic and the transitive causal rule cannot be extracted by other methods. Although non-statistical methods can generate combined causal rules, but are not generating specific rule and relationship strength. One more advantage of our method is that it also generates more specific rule and their strength between indicators. For example, when we run our algorithm on the synthetic-1 dataset, rules are extracted with various properties as lag value (time period after which one affects another indicator), strength and the rate of change of indicators i.e. positive or negative percent change. Actually, the rule *I*_1_ → *I*_3_ is extracted as $(I_{1} , I, 2\% , 1) \Rightarrow \left( {I_{3} , 1\% } \right)$, 113.6, which indicates 2 % change in *I*_1_ inversely effect 1 % change in *I*_3_ after 1 year with 113.6 relationship strength. The results of proposed method are also demonstrated with real world data sets, as described in the following.

To investigate various causal rules in the real world cases, we run the proposed algorithm on the three real world data sets shown in Table 5 for performance evaluation. The proposed algorithm generates various binary, many to one, transitive and cyclic rules, some of the causal rules are reasonable as judged by common sense, shown in Table 8. For example, from the IMF data set, it is found that increases in general government revenue would also increase the volume of exports of goods, increase in growth of general government revenue and gross national saving effect to increase in total investment, and a decrease in government revenue can lead to decreased exports of goods too. Some interesting causal relationships are also extracted in the WTO and World Bank dataset. For example, if crop production of a country is increased, it effects to increase the export of agriculture raw material which helps to improve the economic growth of a country.

### Performance evaluation

This section presents measures for assessing how accurately our proposed method can generate causal rules. The used accuracy measures (Han et al. 2011) are Precision, Recall, Specificity, F-score, Accuracy (recognition rate) and Misclassification rate. We evaluated all measures for proposed, statistical and non-statistical methods compared previously. Binary rules are considered to predict accuracy because this can be generated by all compared methods. Initially we classify the results in two classes as a causal rule (CR) and non-causal rule (NCR). Then, based on the CR and NCR results confusion matrix (TP, TN, FP, FN) is created to evaluate measures shown in “Appendix”. Finally accuracy measures are calculated using TP, TN, FP and FN values. Performance of various methods is evaluated in real world, World Bank dataset for five different scales (numbers of indicators): 10, 20, 30, 40 and 50. Number of target indicators is set to 5 and remain same for all different scales. In Table 10, WBD-10 represents that 10 indicators are considered for causal rule extraction similarly others can be interpreted. Causal rules (some of them) extracted by most of the compared methods are shown in “Appendix”. To indicate extracted causal rules significance appropriate references from previous literatures and documents are given. In Table 10, we can see that the proposed method can achieve higher accuracy and less error rate than all other statistical and non- statistical method for different scales of World Bank dataset.

**Table 10:** Prediction accuracy of proposed, statistical and non-statistical methods on different scales

Accuracy parameters	Proposed method	Li et al. (2013)	Jin et al. (2012)	Silverstein et al. (2000)	Granger causality	Bayesian network
*WBD-10, Rules: 50, CR:16, NCR: 34*						
Sensitivity	0.94	0.81	0.75	0.69	0.69	0.75
Specificity	0.91	0.82	0.74	0.65	0.68	0.79
Precision	0.83	0.68	0.57	0.48	0.50	0.63
F-Score	0.88	0.74	0.65	0.56	0.58	0.69
Accuracy	0.92	0.82	0.74	0.66	0.68	0.78
Misclassification rate	0.08	0.18	0.26	0.34	0.32	0.22
*WBD-20, Rules: 100, CR:38, NCR: 62*						
Sensitivity	0.92	0.84	0.74	0.68	0.66	0.76
Specificity	0.90	0.82	0.74	0.66	0.68	0.77
Precision	0.85	0.74	0.64	0.55	0.56	0.67
F-Score	0.89	0.79	0.68	0.61	0.60	0.72
Accuracy	0.91	0.83	0.74	0.67	0.67	0.77
Misclassification rate	0.09	0.17	0.26	0.33	0.33	0.23
*WBD-30, Rules: 150, CR: 65, NCR: 85*						
Sensitivity	0.91	0.80	0.72	0.63	0.65	0.77
Specificity	0.88	0.81	0.73	0.65	0.66	0.78
Precision	0.86	0.76	0.67	0.58	0.59	0.72
F-Score	0.88	0.78	0.70	0.60	0.62	0.75
Accuracy	0.89	0.81	0.73	0.64	0.65	0.77
Misclassification rate	0.11	0.19	0.27	0.36	0.35	0.23
*WBD-40, Rules: 200, CR: 88, NCR: 112*						
Sensitivity	0.91	0.80	0.68	0.60	0.59	0.70
Specificity	0.89	0.81	0.71	0.63	0.61	0.72
Precision	0.87	0.77	0.65	0.56	0.55	0.65
F-Score	0.89	0.78	0.67	0.58	0.57	0.68
Accuracy	0.90	0.81	0.70	0.62	0.60	0.72
Misclassification rate	0.10	0.20	0.30	0.39	0.40	0.32
*WBD-50, Rules: 250, CR: 112, NCR: 138*						
Sensitivity	0.90	0.79	0.65	0.60	0.57	0.67
Specificity	0.88	0.79	0.67	0.61	0.59	0.68
Precision	0.90	0.75	0.62	0.55	0.53	0.63
F-Score	0.90	0.77	0.63	0.58	0.55	0.65
Accuracy	0.89	0.79	0.66	0.60	0.58	0.68
Misclassification rate	0.09	0.21	0.34	0.40	0.42	0.32

**Fig. 4:**
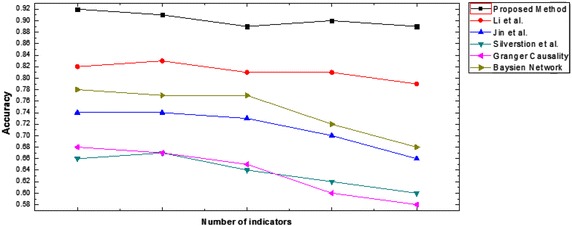
Accuracy curve of various methods on different scales

The accuracy curve for proposed method and the compared methods is shown in Fig. 4. The proposed method can extract causal rules more accurately and performs the best in all different scales. We can also notice when the dataset size increases; the statistical method performance degrades more than non-statistical methods. We regard our proposed method has a stable and good performance accuracy in comparison with the other compared methods.

In summary the comparison results show that the proposed method has high performance and also performs well in terms of all accuracy measures as compare to other compared methods.

### Complexity

The steps defined in an algorithm to make minimum passes over the data. In the first pass, we calculate the growth rate of parameters and its positive, negative or neutral growth rate change value *U, D,* and *Q* are assigned to each parameter to perform the next steps. In the second pass, we calculate the support value and an odds ratio of all the individual parameters together with other parameters for different lag values. Non-zero lag value associations identified from the tests are considered. Associations with insufficient support and odds ratio will be eliminated directly. The cause–effect rules in current pairs can be determined from temporal associations and temporal odds ratio for nonzero lag value. At the end, causal pairs found previously are combined for the next steps to generate transitive, many to one and cyclic rule using basic causal binary rule. To achieve efficiency, all the combinations are not considered as a condition during the generation of other causality rules. Instead, we only investigate the combinations appearing in the data which are related to non-zero lag value. Since such combinations are very small as compared to total combinations, the cost of computation is reduced.

To analyze the performance of the algorithm with respect to time and space complexity, and the number of passes over the data set, we denote the set of parameter *S*, the number of parameters *n*, the length of the time series *t*, the number of extracted pairs *m* and the lag value *l*. The complexity of the method is discussed based on the extraction of binary causal rules in the form of *P*_1_ → *P*_2_ for lag value *l*.

The single parameters are paired and the support is calculated with *O*(*n*) passes over the data set. Each pair combination needs to test for *l* lag values to determine the association and causality, which requires *O*(*n* * *l*) passes. In the process of extracting binary causal relationships, a causal association will be examined on all combinations.

The total number of possible pair combinations *P* is:

19 $$P = \mathop \sum \limits_{n = 1}^{\left| s \right|} \mathop \sum \limits_{m = 1}^{\left| s \right|} \left( {s_{{C_{m} - }} s - m_{{C_{n} }} } \right)$$

So the data set needs to scan as many as *O (Pnl*) times. This way we can conclude the passes over the data set is *O (Pnl*), and the time it takes is *O* (*Pnlt*). Complexity will be substantially reduced by firstly applying the pruning step1 (binary rule generation) before extraction of other relationships.

## Conclusion

This paper proposed a novel method to extract various types of causal relationship like binary, transitive, many to one and cyclic in large time series database. The proposed method is generating more specific rules and their strength which are useful for strategic information. We also defined the concept of temporal odds ratio to categorize temporal association as a causal rule. Experiments have shown that the proposed algorithm can extract single, transitive, combined and cyclic causes from large time series data sets. Additionally, the extracted rules are validated to prove their accuracy and the algorithms have been shown to scale up well with respect to the number of indicators on time series data.

In future, the efficiency of the method can be improved by using fast algorithms of mining association rule. The concept of the algorithm can also be extended to other types of time series. The proposed method can be applied in various social, economic, agriculture domains to generate strategic rules for decision making. The method is also useful to detect the exact cause of fault for the large mechanical system which is monitored by various sensors generating time series data.

## Figures and Tables

**Fig. 5:**
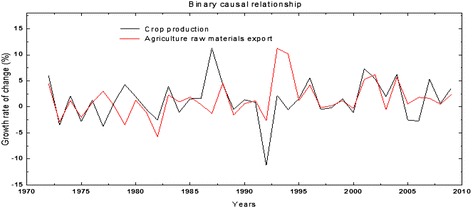
Time series graph for rule CP–(2) → ARME

**Fig. 6:**
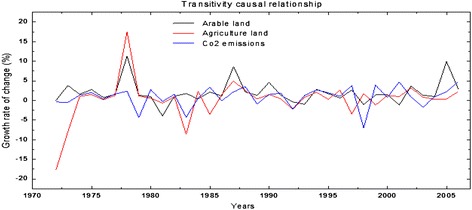
Time series graph for rule AR–(1) → AG–(3) → CO2

**Fig. 7:**
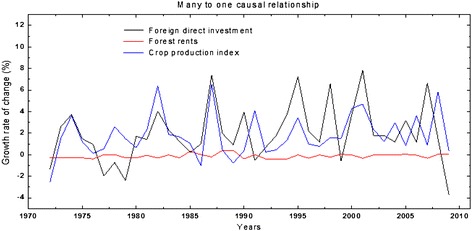
Time series graph for (FDI, FR)–(1) → CPI

**Fig. 8:**
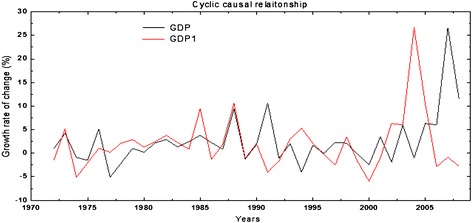
Time series graph for rule GDP ← (2,1) → CY

## Appendix

We can also examine the accuracy of the proposed method through by plotting time series graph between indicators. We have shown time series for four causal relationships. Table 11 shows the growth rate change of parameters for the time period 1972–2009. It represents a value with a lag difference. For example, consider a binary rule CP–(2) → ARME, indicates CP effect ARME after 2 years. In Table 11 value 5.93, shows the growth rate of change of CP in 1972 and 4.35 in the same row shows the growth rate of change of ARME in 1974. Italic values represent the pairs which follow the relationship for a rule. Similarly, we can interpret all entries of other indicators.

**Table 11:** Growth rate change of indicators

Rules	Binary (CP–(2) → ARME)		Transitivity (AR–(1) → AG–(3) → CO2)			Many to one (FDI, FR)–(1) → CPI			Cyclic GDP ← (2,1) → CY		
Year	CP	ARME	ANE	OR	CO2GF	FDI	FR	CPI	GDP	CY	GDP1
1972	5.93	4.35	0	−17.69	−0.4	−1.33	−0.3	−2.56	0.96	−2.63	−1.49
1973	−3.49	−2.92	3.81	−8.06	−0.52	2.58	−0.3	1.5	4.21	2.67	5.12
1974	2.06	1.15	1.56	0.98	1.36	3.72	−0.3	3.62	−0.91	−1.28	−5.11
1975	−2.83	−2.03	2.79	1.51	2	1.47	−0.3	1.2	−1.49	−3.55	−2.06
1976	1.25	0.78	0.73	0.2	0.38	0.95	−0.4	0.11	5.12	0.24	1.02
1977	−3.79	2.98	1.56	1.27	1.63	−1.98	0	0.54	−5.11	−3.24	0.19
1978	0.74	0.23	11.28	17.52	2.36	−0.73	0	2.59	−2.06	−1.67	2.15
1979	4.25	−3.49	1.29	1.12	−4.38	−2.4	−0.3	1.48	1.02	0.97	2.89
1980	1.96	1.26	1.01	0.57	2.74	1.69	−0.3	0.66	0.19	2.65	1.3
1981	−0.54	−1.15	−4	−0.72	−0.27	1.41	−0.06	2.33	2.15	3.65	2.43
1982	−2.54	−5.78	1.09	0.96	1.54	4.03	−0.31	6.37	2.89	1.32	3.79
1983	3.9	2.25	1.73	−8.63	−4.27	2.3	−0.03	1.88	1.3	−1	2.24
1984	−1.06	0.95	0.4	2.32	0.52	1.25	−0.3	1.65	2.43	−1.35	0.87
1985	1.53	1.87	2.12	−3.6	3.36	0.21	0.3	1.04	3.79	10.24	9.4
1986	1.6	0.44	1.14	1.44	−0.11	0.99	0	−1.02	2.24	−3.75	−1.27
1987	11.21	−1.29	8.62	4.99	2.18	7.39	−0.21	6.55	0.87	−0.82	1.82
1988	4.32	4.55	2.13	2.47	3.52	1.96	0.4	0.43	9.4	1.35	10.62
1989	−0.52	−1.57	1.27	0.38	−0.98	0.9	0.4	−0.79	−1.27	−0.77	−1.07
1990	1.33	0.59	4.58	1.5	1.53	3.95	−0.4	0.36	1.82	1.41	2.03
1991	0.85	1.15	1.34	0.41	1.84	−0.53	0	4.08	10.62	−0.76	−4.01
1992	−11.28	−2.68	−0.36	−2.25	−2.21	0.67	−0.4	0.25	−1.07	−0.42	−1.66
1993	2.18	11.22	−1	0.78	1.25	1.77	−0.4	0.45	2.03	−1.51	3.02
1994	−0.55	10.19	2.83	2.09	2.7	3.79	−0.4	1.36	−4.01	−6.12	5.3
1995	1.57	1.22	1.73	0.31	1.94	7.22	−0.03	3.43	1.66	−9.27	2.14
1996	5.57	4.17	0.61	2.59	1.05	2.17	−0.33	0.98	−0.02	−0.33	−0.3
1997	−0.51	−0.17	2.65	−3.49	3.75	1.16	−0.03	0.77	2.3	1.59	−2.44
1998	−0.2	0.22	−1.08	1.78	−7.02	6.56	−0.23	1.58	2.14	1.27	3.46
1999	1.52	1.15	1.38	−1.17	3.92	−0.6	0.03	1.5	−0.3	−5.29	−1.86
2000	−1.13	−0.3	1.48	1.29	0.75	3.74	−0.03	4.29	−2.44	−7.67	−5.93
2001	7.34	5.24	−1.2	0.96	4.7	7.84	−0.33	4.69	3.46	−2.73	−1.01
2002	5.37	6.18	3.61	3.2	0.88	1.78	−0.04	2.36	−1.86	−2	6.3
2003	1.97	−0.54	1.34	0.84	−1.75	1.76	−0.03	1.21	5.93	3.21	6.01
2004	6.2	5.68	0.96	0.28	0.93	1.18	−0.03	2.96	−1.01	−2.27	26.63
2005	−2.55	0.57	9.9	0.39	2.17	3.17	0.03	0.85	6.3	4.45	10.4
2006	−2.74	1.81	2.84	2.27	4.76	1.16	−0.06	3.61	6.01	−0.86	−2.85
2007	5.28	1.63				6.67	−0.33	0.91	26.63	−2.38	−0.86
2008	0.63	0.5				1.44	0.03	5.82	11.4	−0.39	−2.77
2009	3.58	2.39				−3.71	0.03	0.37			

All time series graphs are generated based on the values given in Table 11. Figure 5 shows the time lagged relations between Cereal Production (CP) and Agriculture raw material exports (ARME) with lag 2. A time period where indicators follow the direct relationship for given rule are: {1972, 1973, 1974, 1975, 1976, 1978, 1980, 1981, 1982, 1983, 1986, 1988, 1990, 1991, 1993, 1995, 1996, 1997, 1998, 1999, 2000, 2001, 2002, 2004, 2006, 2008, 2009}. For each time period (say 1974) rule would be interpreted as if the growth of CP has increased in the year 1974 it will increase ARME in 1976. In Fig. 5, time series graph we can observe that parameter satisfied the minimum support and odds ratio which indicates that indicators (CP and ARME) are causally related. Since most of the time, an increase in CP increases ARME, this relationship can be considered as a binary causal (U–U) direct relationship (Table 11).

Figure 6 shows the time lagged transitive causal relationship between AR, AG, and CO2 with lag 1 and 3. Time where indicators follow the relationship for given rule are: {1974, 1975, 1976, 1977, 1978, 1980, 1981, 1982, 1984, 1987, 1988, 1990, 1991, 1992, 1994, 1995, 1996, 1998, 2000, 2002, 2004, 2005, 2006}. For each time period (say 1974) rule would be interpreted as increase in AR in 1974 will increase AG in 1975 which again increases CO2 in 1978. From Fig. 6, we can conclude that the rule satisfied the minimum support and indicators (AR, AG, and CO2) are causally related. Most of the time increase in AR increases AG after 1 year, which again increases the CO2 after 3 years. This rule can be considered as a transitive causal (U–U–U) direct relationship (Table 11).

Figure 7 shows the time lagged many to one relation between (FDI, FR) and CPI with lag 1. Time period follows this relationship can be seen in many to one rule in Table 11 as italic values. In this relation, both indicators FDI and FR together affect CPI after 1 year. In Fig. 7, we can observe that if FDI increases and FR decreases they tend to increase the CPI, i.e. FDI and FR both are the cause of CPI. Indicators follow many to one (combined) causal (U,D)–U relationship. Table 12, shows confusion matrix (TP, TN, FP, FN) values to evaluate accuracy measures and Table 13, represents the causal rules extracted by most of the compared methods.

**Table 12:** Confusion matrix for proposed, statistical and non-statistical methods on different scales

Accuracy parameters	Proposed method	Li et al. (2013)	Jin et al. (2012)	Silverstein et al. (2000)	Granger causality	Bayesian network
*WBD-10, Rules: 50, CR:16, NCR: 34*						
TP	15	13	12	11	11	12
TN	31	28	25	22	23	27
FP	3	6	9	12	11	7
FN	1	3	4	5	5	4
*WBD-20, Rules: 100, CR:38, NCR: 62*						
TP	35	32	28	26	25	29
TN	56	51	46	41	42	48
FP	6	11	16	21	20	14
FN	3	6	10	12	13	9
*WBD-30, Rules: 150, CR: 65, NCR: 85*						
TP	59	52	47	41	42	50
TN	75	69	62	55	56	66
FP	10	16	23	30	29	19
FN	6	13	18	24	23	15
*WBD-40, Rules: 200, CR: 88, NCR: 112*						
TP	80	70	60	53	52	62
TN	100	91	80	70	68	81
FP	12	21	32	42	43	33
FN	8	18	28	35	37	31
*WBD-50, Rules: 250, CR: 112, NCR: 138*						
TP	101	88	73	67	64	75
TN	122	109	93	84	82	94
FP	11	29	45	54	56	44
FN	11	24	39	45	48	37

**Table 13:** Extracted causal rules

Causal rules	References
Transport and travel services → CO2 emission	Stewart et al. (2015)
Electricity production from renewable sources → CO2 emission	Abolhosseini et al. (2014)
Fossil fuel energy consumption → CO2 emission	Mellios et al. (2011)
Household final consumption expenditure → CO2 emission	Tian et al. (2016)
Industry → CO2 emission	Cai et al. (2016)
Electricity production from nuclear sources → CO2 emission	EPA (1970)
Land under cereal production → CO2 emission	EPA (1970)
Air transport, passengers carried → CO2 emission	Stewart et al. (2015)
Livestock production index → CO2 emission	EPA (1970)
Alternative and nuclear energy → CO2 emission	EPA (1970)
Electricity production from nuclear sources → CO2 emission	EPA (1970)
Combustible renewable and waste → CO2 emission	EPA (1970)
Crop production index → CO2 emission	EPA (1970)
Food production index → CO2 emission	EPA (1970)
Oil rents → CO2 emission	EPA (1970)
Forest rents → CO2 emission	EPA (1970)
Cereal production → gross domestic product (GDP)	StatsCan (1971)
Agricultural raw materials exports → gross domestic product (GDP)	StatsCan (1971)
Cereal yield → gross domestic product (GDP)	StatsCan (1971)
Food production index → gross domestic product (GDP)	StatsCan (1971)
Land under cereal production → foreign direct investment (FDI)	StatsCan (1971)
Agricultural land → food production index	FAO (1945)
Arable land → food production index	FAO (1945)
Cereal production → food production index	FAO (1945)
Cereal yield → food production index	FAO (1945)
Permanent cropland → food production index	FAO (1945)
Crop production → food production index	FAO (1945)
Livestock production → food production index	FAO (1945)
Household final consumption expenditure → GDP growth	BIS (2011)
Net trade in goods and services → gross domestic product (GDP)	BIS (2011)
Exports of goods and services → gross domestic product (GDP)	Mehmood (2012) and Ogawa et al. (2016)
External debt stocks → gross domestic product (GDP)	Mehmood (2012)
Final consumption expenditure → gross domestic product (GDP)	Mehmood (2012)
General government final consumption expenditure → gross domestic product (GDP)	Mehmood (2012)
Gross national income → gross domestic product (GDP)	Mehmood (2012)
Gross national expenditure → gross domestic product (GDP)	Mehmood (2012)
Gross domestic savings → gross domestic product (GDP)	Rasmidatta (2011)
Foreign direct investment (FDI) → gross domestic product (GDP)	Li (2005)
Gross domestic product (GDP) → oil rents	Ebeke and Omgba (2011)
Foreign direct investment (FDI) → oil rents	Ebeke and Omgba (2011)
Agriculture → rural population	Enyedi and Volgyes (2016)

Figure 8 shows the time lagged cyclic relations between GDP and CY with lag 2 and 1. Time period follows this relationship can be seen in the cyclic rule in Table 11 as italic values. In this cyclic relation, one more indicator GDP1 values are given which is nothing but the value of GDP after 3 years. Here GDP effect CY after 2 years, which again affect GDP after 1 year, i.e. GDP follows cyclic relation with 3 years delay. In Fig. 8, we can observe that increase in GDP again increases it after 3 years. This cyclic relation follows the cyclic causal U–U relationship.
